# Effects of Physical Exercise on Cardiovascular Diseases: Biochemical, Cellular, and Organ Effects

**DOI:** 10.1155/2015/853632

**Published:** 2015-12-15

**Authors:** Antonio Crisafulli, Pasquale Pagliaro, Alain Cohen-Solal, Andrew J. Coats

**Affiliations:** ^1^Department of Medical Sciences, Sports Physiology Laboratory, University of Cagliari, 09124 Cagliari, Italy; ^2^Department of Clinical and Biological Sciences, University of Torino, Torino, Italy; ^3^Department of Cardiology, Paris Diderot University, UMR-S 942, Lariboisière Hospital, Paris, France; ^4^Monash University, Melbourne, VIC, Australia; ^5^University of Warwick, Coventry, UK

It is well recognised that regular exercise is cardioprotective. On the other hand, sedentariety is an established risk factor for cardiovascular diseases. Furthermore, several clues suggest that exercise capacity is a strong predictor of risk of death from any cause in both healthy subjects and in those with cardiovascular diseases. However, the exact mechanisms through which regular physical activity confers cardiovascular protection are not yet well understood. Exercise probably acts at multiple levels. One possibility is that regular exercise modifies genes expression, thereby changing the production of bioactive molecules such as proteins and enzymes; another potential site of action is on the mechanisms of cardiovascular regulation during effort, which are improved after periods of training; finally, exercise can affect endothelial and platelet functions, thus reducing the risks of atherosclerosis and consequently the risk of infarction and stroke.

This special issue focuses on potential beneficial mechanisms by which exercise operates to confer protection against cardiovascular diseases.

The interesting paper by S. Heber and I. Volf reviews the effects of inactivity and training on platelet functions. In their literature review, they conclude that regular physical activity diminishes or prevents platelet activation in response to acute exercise and that habitual physical activity also positively modulates platelet functions. They also conclude that these effects support the well-recognised relation between exercise and the risk for cardiovascular events, as a physically active lifestyle dramatically reduces cardiovascular mortality.

In another intriguing review, C.-Y. Hsu et al. focus on the important topic of the effects of exercise training on autonomic function in chronic heart failure (CHF). Exercise intolerance is one of the major and disturbing symptoms in these patients. Increased sympathetic tone and decreased parasympathetic activity have been often reported in CHF and these phenomena are associated with a poor survival. Data from this review indicate that participation in exercise training programs induces beneficial effects on autonomic function in CHF patients. They also point out that further research could examine additional aspects of the effects of exercise training in this population, such as the impact on the responses to exercise training in different levels of CHF severity, the possibility that a threshold intensity may be needed to affect cardiac autonomic function, and the type of exercise that should be recommended to achieve the highest positive effects on sympathovagal balance.

In a third review by A. Crisafulli et al., the complex issue of cardiovascular reflexes during exercise is addressed. During exercise, the neural mechanisms controlling the cardiovascular apparatus regulate cardiac output and arteriolar tone in order to counteract the exercise-induced vasodilation due to functional sympatholysis in the working muscle. These cardiovascular adjustments guarantee adequate perfusion to vital organs (the brain and the heart) and to the working muscles as well as adequate washout of exercise-induced by-products. Moreover, these mechanisms prevent excessive increments in blood pressure. In this review, authors summarise neural reflexes operating during dynamic exercise, particularly their interaction. They point out that cardiovascular regulation during exercise is achieved through the contemporary integration and interaction of input arising from motor cortex (central command), skeletal muscle receptors (exercise pressor reflex), and arterial baroreceptors. They also conclude that further research is warranted to better understand how these reflexes interact during effort.

The last interesting review by R. B. Batacan Jr. et al. is about the effects of light intensity activity on cardiovascular risk factors. These authors claim that there is little support for the role of light intensity activity to reduce cardiovascular disease risk factors and that further studies are needed to establish the value of light intensity physical activity in reducing cardiovascular risk factors.

Finally, the research paper by N. G. Rocha et al. aims at evaluating the acute effects of exercise on endothelial functions in early metabolic syndrome. They find that these subjects, despite being free of symptoms, present with an early impairment of endothelial function. Moreover, they put forward the hypothesis that the analysis of some biomarkers changes could be potentially useful to develop preventive measures before the onset of overt metabolic syndrome.

Collectively, these manuscripts confirm that sedentariety is harmful for the cardiovascular system. Moreover, exercise not only exerts positive effect on cardiovascular functions and reduces the risks of cardiovascular diseases (primary prevention), but could also revert cardiovascular disease and play a preventive role in the progression of pathological conditions (secondary/tertiary prevention).

